# New Insights into Polymeric Liquid Crystals and Their Applications

**DOI:** 10.3390/molecules30142997

**Published:** 2025-07-16

**Authors:** A. C. Trindade, J. P. Canejo, P. L. Almeida

**Affiliations:** 1 Atlântica, Instituto Universitário, Fábrica da Pólvora de Barcarena, 2730-036 Barcarena, Portugal; 2 I3N-CENIMAT, Materials Science Department, School of Science and Technology, NOVA University Lisbon, 2829-516 Caparica, Portugal; 3 Mechanical Engineering Department and UnIRE, Instituto Superior de Engenharia de Lisboa, Polytecnic University of Lisbon, 1959-007 Lisbon, Portugal

Polymeric liquid crystals (PLCs) have emerged as one of the most frenetic and interdisciplinary areas of materials science, found at the crossroads of soft condensed matter, chemistry, physics, and engineering. Their ability to combine structural anisotropy with sensitivity to a broad spectrum of stimuli—such as light, temperature, and electric and magnetic fields—positions them as first-choice candidates for the development of multifunctional materials and devices [1]. The last decade has witnessed frenzied activity in the synthesis, characterization, and application of PLCs, and an indicator of this activity is the high number of articles published in high-impact journals. With this Editorial, the authors aim to provide a snapshot of recent progress in this research area, as well as the works published in this Special Issue, and look ahead to future directions based on some of the most significant recent advances in the area.

Recent developments in liquid crystal elastomers (LCEs) targeted high actuation efficiency and low operating thresholds. Lagerwall et al. introduced photoresponsive LCEs from oligomeric precursors, engineered to be active at physiological temperatures. The LCEs demonstrated efficient light-activated actuation independent of heat and, hence, are appropriate for biomedical applications such as light-actuated microfluidics and implantable devices [2]. This work follows the path of existing research regarding the regulation of phase behavior and mechanical response via molecular weight distribution and an oligomer structure. Aside from this research direction, Lin et al. developed dual-responsive LCEs through the integration of azo-functionalized polythiourethanes and polysiloxanes and mechanically interlocked molecules. The resulting systems are extremely robust and self-healing, offering a route to obtaining reconfigurable and durable soft actuators [3].

Programmable shape memory and mechanical response in polymer-dispersed liquid crystal elastomers is another innovation. Rešetič et al. introduced LC droplets in an elastomer matrix, for which there is dual-response functionality-reversible actuation in response to stimuli and shape memory in the absence of forces. Order and elasticity decoupling presents a unique pathway towards designing autonomous systems and smart soft robots [4].

PLCs are increasingly being applied in optoelectronics due to their intrinsic ability to self-assemble into photonic, anisotropic, and birefringent materials. Mysliwiec et al. described a white-light-emitting family of composites comprising PLCs and electric field-tunable photoluminescent dyes. These phase-separated structured composites can be used for future visible-light communication and lighting technologies [5]. Co-related developments include Oton et al.’s recent demonstration of directionality in laser emission via the use of blue-phase photonic crystals, based on polymer-stabilized three-dimensional LC structures [6].

In electro-optics, Singh et al. developed PDLCs containing carbon nanotube and graphene oxide additives. The resulting composites provided improved dielectric properties, higher contrast ratios, and reduced response rates (critical to the integration of smart windows and privacy glass) [7]. Similarly, Vignolini et al.’s research on switchable whiteness in LC polymer networks is the manifestation of onset dual-phase devices with transparency and reflection control through polymer confinement [8].

Azobenzene-functionalized PLCs continue to be valuable building blocks for light-switched actuation devices. Rešetič et al. investigated selectively deuterated mesogens in LCE networks for high-resolution manipulation of the interplay between the absorption of light and phase transition dynamics. This facilitated next-generation mechanical reconfiguration towards adaptive optics and soft robotics applications [4]. Meanwhile, Beeckman et al.’s research on off-axis reflective holograms through photo-alignment of chiral LCs provided a direction for high-resolution, low-power display technology [9].

Additionally, research on the 3D nanoprinting of LC-based photonic devices with large, relevant Kerr nonlinearities has been led by Wiersma et al. in the field of nonlinear optics. Such soft photonic devices, processed through two-photon polymerization, provide modulation of spatial refractive index on scales of sub-micron length—required for on-chip photonic circuitry [10]. Concurrent work focusing on modulating light–matter coupling in LCEs with embedded chromophores, as carried out by Li et al., is directed toward third-order nonlinear optical responses [11].

Charge transport and semiconducting characteristics in PLC systems have been extensively researched as well. Singh et al. researched columnar phases as active semiconducting channels in OFETs, their anisotropic transport, and morphological stability [12]. Laschat et al. also researched MR-TADF liquid crystals with narrowband emission of the mesophase itself, a new OLED technology resource [13].

Hybrid composites of PLCs and 2D materials or magnetic particles are still in vogue as a research topic. Yoon et al. researched PLC composites made of aligned MXene nanosheets with humidity-induced actuation and enhanced thermal conductivity [14]. Clark et al. focused on another related area of work, researching the magneto-optical response of ferromagnetic LCs. They found emergent behavior under rotating fields, and their research contributed towards the development of magnetically tunable devices [15]. One of the most fascinating current research areas is the synthesis of ferroelectric nematic phases and their polymer counterparts. A theory concerning PLC analogs with spontaneous polarization was developed by Mandle in a study on polar nematic order [16].

At the manufacturing level, techniques like laser patterning, field-assisted alignment, and multi-material 3D printing are enabling ultra-high-precision control of PLC domain orientation. Laser writing of nanogroove patterns for planar alignment of nematics in intricate geometries was the subject of research by Muševič et al. [17]. In parallel, in Sánchez-Somolinos et al.’s research, it was found that PLC actuators enable dynamic actuator shape reconfigurability—an approach in harmony with soft reconfigurable robotics and shape-morphing systems [18].

In our opinion, three prospective research areas are particularly relevant. The first concerns the development of green PLCs from bio-derived building blocks such as cellulose, chitin, or xanthan, a promising field in environmental sensing, biodegradable photonics, and environmentally friendly electronics. Godinho et al. have led such developments to demonstrate morphochromatic cellulose-based sensors with anisotropic optical responses [19].

Secondly, quantum photonics based on LC is an emerging field. Humar et al. have demonstrated entangled photon pairs produced from ferroelectric LC cells and proposed their application in quantum encryption and interfacing matter with light at the nanoscale [20].

Thirdly, topological engineering in PLCs is progressively becoming recognized as a viable method for the stabilization of a low-power optical memory state and metastable texture. The work carried out by Kos et al. on multistable polar textures is in line with research area, and it is also applicable to non-volatile information storage and holographic display technology [21].

The articles published in this Special Issue reflect and expand upon the current landscape of polymeric liquid crystals, addressing key scientific challenges and advancing our understanding of materials properties, novel functionalities, and potential technological applications. The study by Mattsson et al. provides a thorough investigation of the interplay between structural anisotropy and mechanical deformation, helping to elucidate the origins of auxetic behavior in LCEs. Their experimental analysis (combining dielectric spectroscopy, calorimetry, and rheology) demonstrates how strain induces configurational constraints that coincide with out-of-plane mesogen rotations, contributing to the growing body of knowledge on stimuli-responsive LCE mechanics [22]. Amaral’s reflective review delivers a multidisciplinary perspective grounded in decades of work across soft matter, lyotropics, and biomolecular systems. While less focused on new data, this article plays a critical conceptual role in reminding the field of the importance of structural scaling laws, interdisciplinarity, and fundamental insights as a driver of applied research in complex soft materials [23]. Hwang et al. contribute with an original approach to chemical sensing. Their development of alcohol sensors based on cholesteric liquid crystals and carboxylate polymers enhances our understanding of photonic bandgap tuning for selectivity and sensitivity in LC-based detection systems. By demonstrating how UV curing affects performance, they address the need for simple yet effective analyte discrimination, such as discriminating between methanol and ethanol [24]. Wang et al. present an innovative bilayer PDLC structure with enhanced optical and anti-counterfeiting capabilities. Their materials engineering approach, made possible through nanoparticle doping and multilayer design, exemplifies how structural design can be leveraged to tailor electro-optical and functional responses [25]. Tokita et al. report the synthesis of side-chain liquid crystalline polyacrylates incorporating bridged stilbene mesogens, achieving nematic phases near room temperature and notable birefringence. Their polymers exhibit aggregation-induced emission and cybotactic cluster formation, offering valuable insights into π-conjugated SCLCPs and their potential in luminescent and anisotropic optical materials [26]. Kikuchi et al. present a transparent nano-PDLC system utilizing a ferroelectric Smectic A (SmAF) liquid crystal to enhance birefringence memory effects. By incorporating highly polar SmAF materials into sub-micron phase-separated structures, they achieved improved electro-optic response and retention of induced molecular orientation after field removal. This work highlights the potential of SmAF LCs in low-voltage, memory-type displays and tunable photonic devices, advancing the functionality of PDLCs beyond conventional nematic systems [27]. Choi et al. present a microlens array-imprinted polymer-dispersed liquid crystal (PDLC) film developed via a low-cost spin-coating method to enhance OLED outcoupling efficiency. Their flexible, high-haze PDLC structure achieves a 37.5% improvement in light extraction without compromising electrical performance. This work demonstrates the practical integration of soft lithography and LC-based scattering layers for flexible optoelectronics, pointing toward scalable roll-to-roll manufacturing of next-generation OLED displays [28]. Wie et al. report a hybrid top-down/bottom-up strategy for fabricating highly aligned liquid crystalline polymeric fibers using spatial confinement. By thermally oligomerizing LC monomers within microchannels and subsequently photopolymerizing them, the authors demonstrate the controlled transition from spherical micelles to linear, defect-free nematic fibers. This confinement-driven self-assembly offers an alternative to high-shear or electrospinning techniques and paves new pathways for the scalable production of anisotropic LC fibers for soft actuators and responsive materials [29]. Finally, Zakaria et al. present a detailed structure–property investigation of Schiff-base ester liquid crystals featuring pyridyl and phenyl heterocycles. Through experimental mesophase characterization and complementary DFT analysis, the authors demonstrate how mesogenic core orientation and terminal alkyl chain length critically influence mesophase type and thermal stability. The study identifies dipole moment, planarity, and π–π stacking potential as key predictors of nematic versus smectic behavior, offering valuable design rules for tailoring new mesogenic architectures with tunable electro-optic properties [30].

Collectively, these papers present a coherent picture of the developments in polymeric liquid crystal research, from providing a fundamental understanding of mechanical relaxations and auxetic behavior in elastomers to innovative applications in sensing, photonics, soft actuation, and smart interfaces. They also demonstrate the importance of coupling molecular design with advanced processing techniques (such as electrospinning, surface grafting, or microfluidic templating) to enable next-generation materials. Future work in this area will undoubtedly build on the insights offered here, exploring hybrid architectures, multiresponsive functionalities, and scalable manufacturing methods. Polymeric liquid crystal science is growing and developing. By integrating dynamic molecular self-assembly with mechanical response and optical function, PLCs offer an unprecedented versatility of material platform. The research documented in this Special Issue not only heralds recent achievements but also predicts the exciting future prospects of PLCs as enabling technologies for future generations of smart, responsive, and multifunctional systems.
